# Neuroscience of Adolescent Anorexia Nervosa: Implications for Family-Based Treatment (FBT)

**DOI:** 10.3389/fpsyt.2020.00418

**Published:** 2020-05-21

**Authors:** Roger Mysliwiec

**Affiliations:** New Zealand Eating Disorders Clinic (NZEDC), Auckland, New Zealand

**Keywords:** eating disorders, anorexia nervosa, family-based treatment (FBT), neuroscience, adolescence

## Abstract

Over the past 20 years significant progress has been made to elucidate some of the neurobiological underpinnings of the development and maintenance of anorexia nervosa and their possible implications for treatment. There is increasing evidence supporting the notion that anorexia nervosa shares neurobehavioral patterns with anxiety disorders and involves reward processing aberrations and habit formation. There is consensus for the need of early intervention to ameliorate the effects of starvation on the adolescent brain and the effects of illness duration on neurodevelopment. Family-based treatment (FBT) is the first line evidence-based treatment for adolescents with anorexia nervosa achieving sustainable full remission rates of over 40%. FBT has an agnostic treatment approach and its mechanisms of change have until now not been fully understood. To help fill this gap in theoretical understanding, this paper will provide a review of the treatment model of FBT through a neuroscientific lens. It argues that FBT is well designed to address the implications of current key findings of the neuroscience of anorexia nervosa and that it is also well aligned with the current understanding of neuroscience principles underpinning therapeutic change. The paper supports the perspective that FBT utilizes principles of parent facilitated exposure response prevention. It concludes that an integration of a neuroscience perspective to the provision of FBT will assist the clinician in their practice of FBT.

## Introduction

Anorexia nervosa (AN) is a serious psychiatric illness with a peak age of onset during adolescence (1) and is associated with high rates of chronicity and the highest mortality rate of psychiatric disorders, alongside substance use disorders (2, 3). Longer illness duration is associated with a more chronic course of illness and poor prognosis (4). Self-induced starvation leads to acute and chronic states of malnutrition with potential life-threatening physical complications as well as neurocognitive sequelae. The causes of AN are of a complex nature and neuroscience has begun to elucidate some of the likely underpinning factors contributing to its aetiology (5).

The need and importance for early intervention has been well established (6). Outcomes of psychological specialist individual treatments are generally poor with full recovery rates between 22% and 28% (7). Recovery rates of family-based treatment (FBT) for adolescent AN are far superior to individual treatment with rates of full recovery of 47% at 12-month follow-up versus 23% at 12-month follow-up for adolescent-focused therapy (AFT) (8). FBT is effective for adolescents up to the age of 18 with an illness duration of up to 3 years.

There have been calls to develop treatments that specifically target some of the underpinning factors contributing to aetiology and maintenance of psychiatric illnesses and move away from treatment approaches that are agnostic to illness aetiology. The National Institute of Mental Health (NIMH) has developed a framework of Research Domain Criteria (RDoC) based on observable behavior and its underpinning neurobiology with the aim to help develop precision medicine for psychiatry (9). This has over the past few years also increasingly been applied to research of eating disorders (10) and is being translated into novel treatment concepts (11, 12). For instance, Maudsley Anorexia Treatment for Adults (MANTRA) has been developed based on empirical research and its treatment targets are aimed to address putative predisposing and maintaining factors like a perfectionistic rigid thinking style as well as an emotionally avoidant interpersonal style. However, in RCTs MANTRA has not outperformed any of the other psychological treatments for adult AN like Cogntive Behavior Therapy Enhanced (CBT-E) or Specialist Supportive Clinical Management (SSCM) (13). Most of the other treatment approaches that have been developed based on neuroscientific findings such as repetitive Transcranial Magnetic Stimulation (rTMS) and Deep Brain Stimulation (DBS) are still in their experimental stage with reasonably small case series even though they are starting to show promising results (14). It is critical that academic centers continue to develop and test treatment models based on neuroscience with the ultimate objective to match a targeted treatment approach with the individual's presentation.

Understanding the neurobiology of AN also holds potential for a variety of treatment applications in relation to existing psychological therapies (15). In order to bridge the gap between research and practice this paper undertakes a review of manualized FBT through a neuroscientific lens. It needs to be acknowledged that the scope of the paper is necessarily focused in its description of the neurobiology of AN.and does for instance not explore models focussing on abnormal neurotransmitter systems (16) or dysregulated frontostriatal systems (17). It will first provide a synopsis of the key tenets of FBT before describing pertinent aspects of the neuroscience of AN with reference to FBT such as the effects of malnutition on the adolescent brain, anxiety as a core feature of AN, and reward processing aberration and habit formation. This will be followed by a discussion of general neuroscience concepts underpinning fear-based learning, habit formation and new learning through neuroplasticity and their relevance to FBT treatment of AN. The conceptualisation of FBT as parent facilitated exposure response prevention (ERP) therapy (18) will be discussed arguing the importance of the need for parent facilitation of ERP in light of the disabling effects of a state of high anxiety in the context of malnutrition for the adolescent brain.

The paper concludes that FBT does address the salient key findings of the neuroscience of AN and that its interventions map well on these neuroscientifically based concepts and models. It argues that it is important that clinicians providing FBT are aware of neuroscience findings of AN and have a general understanding of applied neuroscience informed principles to learning and facilitating change, especially in the context of treatment of anxiety.

It concludes that an integration of a neuroscience perspective to the provision of FBT will assist the clinician in their practice of FBT and will also help shift the parents' perspective from a stigmatised view of the illness to an easily understood biological basis, thereby increasing their motivation and perseverance in helping their child recover.

## Key Tenets of FBT

FBT is theoretically agnostic with no assumptions about the origin of the disorder (19). It has a strict focus on what can be done to alleviate symptoms. Treatment consists of three phases over approximately 1 year. Core tenets of FBT are the use of externalization with separating the illness from the adolescent and the use of the parents as a resource and empowering them to be the agent of change, with no blame directed to either the parents or the ill child/adolescent. Parents are empowered to be in charge with the therapist actively mobilising a sense of urgency in the parents for the need to address the anorexic behavior and focus on weight recovery, without delay.

The therapist is tasked to support the parents to manage and help regulate the extreme anxiety felt by the adolescent when faced with having to eat more. The therapist emphasizes the need for the parents to be on the same page in order to ensure predictability and consistency. The parents are tasked to help manage the anxiety of the adolescent, maintain sight of the needs and importance to adhere to the goals of full recovery of their adolescent. FBT's use of externalization of the illness helps the parents to maintain a supportive, caring relationship with their child.

## Effects of Malnutrition on the Developing Adolescent Brain

Cerebral gray and white matter deficits due to malnutrition in adolescents have been well established (20). Bernardoni et al. (21) have been able to demonstrate that reductions in gray matter volume and cortical thickness are reversible with full weight recovery. Adolescence is a time of significant change in the brain leading to unstable networks. In particular, the maturation of the prefrontal cortex and its executive ability to inhibit impulses of the limbic system is significantly delayed. (22) These neurodevelopmental changes during adolescence involve synaptogenesis and neural pruning and require myelination of neurons permitting faster neurotransmission. This process is linked with the development of abstract thinking, executive function and decision making and emotion regulation. This means that starvation with restricted fat and carbohydrate consumption will have a significant effect on the development of complex neural networks. Recent studies in AN have identified alterations in a range of white matter structures in the limbic system associated with anxiety, body image and cognitive functions (23). These effects on the brain occur at a period of high instability of developing networks. It is unclear yet to what extent these alterations will be reversible but it is likely that faster and full weight recovery will be of benefit.

As much as the need for full weight recovery and the risks of malnutrition might appear obvious to both parents and clinicians, having a deeper understanding and appreciation of the actual impact of malnutrition on the brain, which is less apparent than the more visible effects of malnutrition on the physical body, is of great benefit, because it provides a strong clinical rationale to achieve full weight restoration rather than being satisfied with a weight level that might be sufficient to allow the adolescent to function at a greater capacity physically but does not address the impact on adolescent brain development. This issue of definition of full weight recovery tends to often become a challenge during phase 2 of FBT and the adolescent themselves can also be more receptive to a neuroscientific rationale on the effects of brain functioning.

## Anxiety as a Core Feature of AN

AN is characterised by the experience of very high levels of anxiety in the adolescent in the context of eating. AN and anxiety disorders have a genetically overlapping phenotype with a high incidence of premorbid and comorbid anxiety disorders (24, 25), elevated traits of harm avoidance and trait anxiety (26). This has led to the development of aetiological models centering around the role of anxiety in AN (27, 28). Strober's model posits a heightened sensitisation to fear stimuli leading to the development of rapid pathological fear conditioning. This predisposition to fear conditioning leads to behavioral avoidance with increased resistance to fear extinction learning (28, 29).

Set-shifting deficits (30, 31) are found in adults with AN and tend to persist after full weight recovery but have not been found in adolescent AN indicating that illness duration could play a role highlighting the need for early intervention. High obsessionality has also been identified as a predisposing trait and moderator, adversely affecting treatment outcome and requiring longer treatment (32).

Individuals with predisposing traits of anxiety tend to feel easily overwhelmed, which will be further exacerbated by the state of starvation. The adolescent suffering from AN will try to ignore the starvation induced stress signals in order to be able to continue to pursue their goal of weight loss. This conflict between a cognitively based top down motivation to want to lose weight and the inherent biological need of body and brain to have its nutritional energy needs met will create an ongoing internal dissonance, which in itself is causing increasing stress for the adolescent (33). The implicit effects of starvation on brain and body as well as the acute effects of anorexic anxieties create a permanent state of ongoing heightened levels of stress and anxiety with all its neuroendocrinological effects including chronically elevated cortisol levels. This chronic heightened state of anxiety leads to further fear sensitisation and further impacts on the development of the cortico-limbic pathways required for development of emotion regulation.

It can be very helpful to describe to parents and adolescent how this interplay of factors exacerbates the severity of the anxiety and disadvantages the adolescent, rendering them incapable of reversing the negative cycle of anxiety and avoidance without parental help.

## Reward Processing Aberration and Habit Formation

There have been a number of functional neuroimaging studies (34, 35) demonstrating reward processing aberrations with an underactive “bottom-up” processing of interoceptive pleasure experience in combination with overactive cognitive inhibitory control (27). Reward processing and fear-based learning are fundamental core learning patterns essential for survival that are prone to lead to chunking of motivation behaviors, i.e., habit formation. Automatic and habitual cognitive bias towards AN related preoccupations and behaviors leads to further neural progression and entrenchment of these behaviors. These findings have led to the development of reward centred models of AN (36–39), which describe a process from initial “liking” the consequences of weight loss to “wanting” to pursue this goal further, and then a progression to habit learning over time. This correlates with a shift from behavior being encoded in neural circuits involving amygdala, ventral striatum and orbitofrontal cortex, to those of the dorsal striatum and dorsal prefrontal cortex. These habit learning behaviors are being maintained by the strong reward of fear relief and therefore become even more quickly entrenched and increasingly difficult to change the longer they persist. However, early in the development of AN habits should be less entrenched and the dorsal striatum circuits less engaged (40). This is a strong rationale for the necessity to address the behaviors maintaining AN, which the clinician can use to elicit the same sense of urgency with the parents to challenge anorexic behavior early on to prevent the development of entrenched cue-stimulus learning.

## General Neuroscience Concepts Underpinning Learning Theory

There are a number of fundamental neuroscientific principles underpinning fear-based learning, habit formation and new learning through neuroplasticity that are relevant to the treatment of AN. A highly aroused anxious state involves an inability to utilize the left prefrontal cortex to down-regulate emotions and instead is entirely directed towards immediate threat avoidance (41, 42). Fear-based learning is very fast (just one single synapse from the thalamus to the amygdala), which is very effective to promote survival but in the context of the AN leads to overwhelming distress for the adolescent when exposed to food. Understanding these underpinning neuroscience concepts can help the parents appreciate that in the actual moment of having to eat, their child will feel too overwhelmed with fear and will try to avoid eating even though at other times they might genuinely state that they want to recover.

This can provide compassion and understanding in the parents for their child and at the same time a recognition that they, at least initially, need to be the agent of change to facilitate new behavior.

The cost of fear-based learning is closed loop neural activation of entrenched patterns of avoidance (43, 44). Repetition of behavior strengthens synaptic connections through long-term potentiation (LTP) from α-amino-3-hydroxy-5-methyl-4-isoxazolepropionic acid (AMPA) to N-methyl-D-aspartate (NMDA) glutamate receptors (45), which is particularly relevant for the development of habits. This implies that not engaging in change behavior further strengthens the neural connections underpinning the AN behavior resulting in further entrenchment. Unlearning of behavior is challenging and requires multiple repetitions of new learning experiences in order to build new neural connections. Neuroplasticity is enhanced through new learning and ongoing neural activation results in increased neural wiring (46). The concept of the “window of tolerance” provides an understanding that new learning requires a certain degree of safety to minimize feeling overwhelmed and out of control (47–49). This understanding of the importance of a sense of safety as an essential foundation for exposure work can enhance the parental effort to try to remain calm and compassionate with the adolescent to ensure a sense of safety. Explaining these basic concepts to the parents can emphasize the need to be persistent and consistent in reshaping behavior. It also provides hope that there are known mechanisms of neural change.

## Parent Facilitated ERP as a Therapeutic Mechanism of FBT

ERP is the treatment of choice for both OCD and anxiety disorders. It is now understood that extinction processes largely rely on the development of new inhibitory learning pathways through new positive associations with the original stimuli as opposed to just the habituation to fear stimuli (50). This kind of learning requires multiple supported repetitions and is highly context specific and is based on the development of left prefrontal cortex control over the fear circuit (amygdala), memory retrieval (hippocampus), and reward- and habit-related processes (basal ganglia) (51).

Steinglass et al. (52) conducted a small study using ERP to address anticipatory anxiety with weight restored patients with AN in an inpatient setting, which was moderately successful. Hildebrandt et al. (18) argued that FBT can be conceptualised as a form of parent facilitated ERP therapy. He and his team have subsequently developed exposure-based FBT (FBT-E), which includes a coaching manual and also a range of submodules (53). While his preliminary findings show modest improvements they are difficult to generalise due to low numbers of participants and the fact that it was not compared with standard FBT.

It is a plausible concept to view FBT as a form of parent facilitated exposure, utilizing extinction processes with facilitation of inhibition learning through the development of new behaviors (54). The neuronal pathways required to establish new inhibitory learning are still under construction in a developing adolescent brain and, in a state of malnourishment and heightened anxious arousal will not have an opportunity to develop sufficiently unless parents facilitate this process of preventing an avoidance response in order for inhibitory learning to become more permanently encoded. From a neuroscience perspective one could argue that FBT is designed to mobilise the capacities of the joined prefrontal cortices of the parents, based on the assumption that the adolescent, due to the nature of the illness, is not in a position to make sound decisions while overwhelmed with a state of anxiety. Introducing the idea that the adolescent is feeling too overwhelmed by anxiety to think clearly and make good decisions can help the parents understand that some of the less acceptable behaviors associated with the illness are not deliberately disobedient or manipulative but rather a response to extreme fear. Supporting the relationship of the parents with their child activates attachment behavior, which provides the empathy, emotional warmth, and safety, which are the foundation of successful exposure challenges.

## Summary and Discussion

This paper proposes that an understanding of a neuroscientific model of AN can enhance the clinician's practice of FBT and may help explain to both practitioners and families why certain principles and practices inherent in FBT are effective. The provision of psychoeducation on the neuroscience of AN can help families shift away from a perspective of stigmatised illness with attributions of blame, to accepting the biological basis of AN and thereby assist with the process of externalization of the illness (55). Understanding the malleability of its biological causes can also increase the parents' motivation and perseverance to ensure that they help their child to achieve full recovery (56). It also validates the subjective experience of the adolescent of high distress and fear and thereby can give them a sense that their experience is viewed as “real” rather than just a reflection of insensible resistance. The extent of the adolescent's fear response provides a clear rationale why the parents need to be in charge to help the adolescent tolerate exposure. The concept of the window of tolerance for learning provides an understanding for the parents of the need to be calm and to provide a sense of safety for their child in order to meet the challenge of exposure to eating more. Having a true understanding of the basis of the distress of their child can invite compassion and understanding from the parents without them losing sight of the need to ensure weight recovery and extinction of anorexic behaviors. Appreciating the vulnerable and highly susceptible state of the developing adolescent brain provides a strong rationale, not only for rapid and full weight recovery but also for the need to explicitly address behavior maintaining AN anxieties. This can address the development of habit formation and risk of entrenchment of behavior with its adverse effect on prognosis. ERP therapy is the treatment of choice for OCD and other anxiety disorders. It requires multiple repetitions of exposure to the feared object with response prevention leading to the development of new inhibition learning. The concept of parent facilitated ERP maps well onto FBT. The core tenets of FBT of parental empowerment, use of externalization of the illness and emphasis on attachment and connection with the adolescent through compassion and empathy provide a framework within which the objectives of ERP can be achieved. FBT should not be conceptualised as just another form of ERP but it can be helpful for both clinicians and parents to understand the principles and theoretical underpinnings of ERP as a likely therapeutic mechanism of FBT. Further study of extinction learning in the context of treatments of AN (57) may hold the potential to refine therapeutic mechanisms and thereby hold the potential to improve its efficacy and thereby treatment outcomes.

The past 20 years of research have not only established and confirmed the efficacy of manaualized FBT but have also increasingly provided more data to develop neuroscientific models of the aetiology and maintenance of AN and to improve our understanding of the mechanisms by which treatment works. The task is now to integrate that knowledge into existing therapies like FBT.

## Author Contributions

The content of this paper is solely the responsibility of the author.

## Conflict of Interest

The author declares that the research was conducted in the absence of any commercial or financial relationships that could be construed as a potential conflict of interest.
